# Effects of Oxidized Soybean Meal and Oxidized Soybean Oil on the Muscle Oxidative Stability, Flesh Quality, Amino Acid Profile, and Fatty Acid Profile of *Megalobrama amblycephala*

**DOI:** 10.3390/antiox13111356

**Published:** 2024-11-06

**Authors:** Yangyang Huang, Xiufei Cao, Wenbin Liu, Guangzhen Jiang, Aimin Wang

**Affiliations:** 1 College of Animal Science and Technology, Nanjing Agricultural University, Nanjing 210095, China; 2018105049@njau.edu.cn (Y.H.); cxf@njau.edu.cn (X.C.); wbliu@njau.edu.cn (W.L.); 2 College of Marine and Biology Engineering, Yancheng Institute of Technology, Yancheng 224051, China; blueseawam@ycit.cn

**Keywords:** *Megalobrama amblycephala*, oxidized soybean meal, oxidized soybean oil, oxidative stability, flesh quality, amino acid profile, fatty acid profile

## Abstract

This study aimed to investigate the effects of oxidized soybean meal and oxidized soybean oil on the muscle oxidative stability, flesh quality, amino acid profile, and fatty acid profile of blunt snout bream *Megalobrama amblycephala*. Oxidized soybean meal and oxidized soybean oil were obtained from fresh soybean meal (FSM) and fresh soybean oil (FSO) by heating. In the experimental diet, the proportions of oxidized soybean meal (OSM) and oxidized soybean oil (OSO) were 30% and 4.19%, respectively. The feeding trial was conducted for 8 weeks. The findings revealed that both OSM and OSO reduced glutathione peroxidase (GSH-Px), superoxide dismutase (SOD), catalase (CAT), hardness, chewiness, and oxymyoglobin (OxyMb) and elevated the content of malondialdehyde (MDA), protein carbonyl (PC), and metmyoglobin (MetMb) in the muscle. OSM notably decreased the content of muscle essential amino acids (EAAs), nonessential amino acids (NEAAs), delicious amino acids (DAAs), and total amino acids (TAAs) compared with CON and OSO. Compared with CON and OSM, OSO significantly reduced the content of elaidic acid (C18:1n9t), linoelaidic acid (C18:2n6c), polyunsaturated fatty acids (PUFAs), ω-6 PUFAs, and the ratio of ω-6/ω-3, while stearic acid (C18:0), γ-linolenic acid (C18:3n6) and saturated fatty acids (SFAs) were significantly elevated. In conclusion, this study demonstrated that both OSM and OSO negatively impacted muscle antioxidant capacity and flesh quality. Moreover, OSM adversely affected the amino acid profile of the muscle, while OSO impaired the fatty acid profile.

## 1. Introduction

Lipids not only enhance the energy content of feed but also provide essential fatty acids and phospholipids and facilitate the absorption of fat-soluble vitamins [1]. However, fats are prone to oxidation when exposed to oxidants or oxidative conditions during transportation, processing, and storage. The adverse effects of oxidized fats on animal quality have been well documented. For instance, Arbabi [2] reported that oxidized soybean oil reduced egg production and egg mass in laying hens. The impact of oxidized fats on fish quality has also been extensively studied, including research on channel catfish *Ictalurus punctatus* [3], Amur sturgeon *Acipenser schrencki* [4], and hybrid grouper (♀ *Epinephelus fuscoguttatus* × ♂ *Epinephelus lanceolatus*) [5]. Protein, like fat, is a primary target of oxidation. Protein oxidation, which involves covalent modification by active substances or reaction with secondary products of oxidative stress, leads to structural changes and nutrient loss [6]. While current studies on oxidized proteins focus mainly on food, research in the feed industry is still relatively limited. Existing studies on the effects of oxidized proteins on animals mainly address growth performance and antioxidant capacity [7,8], with little attention to animal quality.

Fish is considered a healthier food than red and processed meats because of its high-quality proteins and polyunsaturated fatty acids (PUFAs), which can reduce the risk of cardiovascular diseases [9]. Aquaculture has become the primary way to meet the increasing demand for fish, playing a crucial role in ensuring global food security. Fish require higher protein levels in their feed than terrestrial animals [10]. For this reason, we suspect that the oxidation of protein ingredients may have significant effects on fish, such as reducing the nutritional value of the fish muscle. Moreover, there is a lack of comparative studies on the effects of protein oxidation and fat oxidation on fish quality. Only one study reported the effects of oxidized corn protein and oxidized corn oil on oxidative stress and granulosa cell apoptosis in the ovaries of laying hens [11].

Blunt snout bream, a traditional Chinese freshwater herbivorous fish, is favored by consumers for its fast growth, high disease resistance, and high carcass yield. In 2022, the annual production of blunt snout bream exceeded 700,000 tons [12]. Herbivorous fish can effectively utilize plant proteins and oils, which is significant for ensuring a food supply for humans [13]. Soybean meal and soybean oil are the most popular plant-based protein ingredient and fat ingredient, respectively, and the most heavily used protein ingredient and fat ingredient in blunt snout bream commercial feeds. Currently, the ratios of soybean meal and soybean oil in the feed formulation for blunt snout bream are approximately 30% and 4%, respectively [14,15,16]. Understanding how oxidized soybean meal affects fish quality and comparing it to the effects of oxidized soybean oil is crucial. Therefore, this study aimed to investigate the effects of oxidized protein and oxidized fat on the growth performance, muscle oxidative stability, flesh quality, and amino acid profile of blunt snout bream. Drawing on previous studies and commercial feed production processes [11,17], we used heat treatment to oxidize fresh soybean meal and soybean oil.

## 2. Materials and Methods

### 2.1. Experimental Design

Fresh soybean meal and fresh soybean oil were supplied by Yihai Kerry Co., Ltd. (Nanjing, China). We first degreased the soybean meal sufficiently with methanol and then placed it in a cold place to dry. Oxidized soybean meal was obtained by heating fresh soybean meal for 15 h at 100 °C. Oxidized soybean oil was obtained in the same manner as the oxidized soybean meal. The degree of oxidation of soybean meal and soybean oil was determined, as shown in Table 1 and Table 2. The acid value (AV) and peroxide value (POV) were determined using titrimetry according to China National Standards GB 5009.229-2016 [18] and GB 5009.227-2016 [19], respectively. Contents of protein carbonyl and free sulfhydryl were determined according to the method described by Huang [20]. The formulation of the experimental diets is presented in Table 3. The preparation of experimental diets was based on a previous method [21]. The prepared particles were dried and stored at −20 °C for later use.

The experimental fish were purchased from a local farm in Wuhan. They were first domesticated in an outdoor pond for a period of two weeks to acclimatize to the aquaculture environment, during which they were fed a commercial diet (30% crude protein, 6% crude fat) from Tongwei Group China (www.tongwei.com, 20 October 2024). Fish in good health and similar size (average initial weight:14.37 ± 0.13 g) were selected and equally distributed into 12 pond nets (2 m × 1 m × 1.5 m, length–width–height), 12 fish per net. Throughout the 8-week feeding trial period, the fish were hand-fed to apparent satiation with experimental diets three times a day at 8:00, 12:00, and 16:00. Dissolved oxygen remained >5.0 mg/L; the temperature of water ranged from 28 to 34 °C; pH was 7.1 to 7.4; and total ammonia nitrogen and nitrite were maintained lower than 0.4 and 0.01 mg/L, respectively.

### 2.2. Sampling

After the end of the feeding trial, all fish were fasted for 24 h, adequately anesthetized with MS-222 (20 mg/L), and then sacrificed by a sharp blow to the head. Fish in each treatment were weighed and counted to calculate the growth performance index. After removing the scales and skin, the dorsal muscle was cut with a sharp knife. Muscles of about 2 mm cross-section thickness were taken and stored in 4% paraformaldehyde for morphometric analysis. A piece of dorsal muscle was placed on ice, and the texture was tested within 24 h. The other muscle samples were collected and quickly placed in liquid nitrogen for subsequent analysis.

### 2.3. Growth Performance

The final body weight (FBW), survival rate (SR), average daily feed intake (ADFI), and weight gain rate (WGR) were calculated according to the following formulas:

Final body weight (FBW, g) = total final weight/total number

Survival rate (SR, %) = 100 × (Final fish number/Initial fish number)

Average daily feed intake (ADFI, g) = Total feed intake/(fish number × experimental days)

Weight gain rate (WGR, %) = (final body weight − initial body weight) × 100/initial body weight

### 2.4. Muscle Chemical Composition Analysis

The crude protein, crude fat, moisture, and ash contents of muscle were determined according to the methods described by the Association of Official Analytical Chemists [22].

### 2.5. Determination of Muscle Antioxidant Indexes

To analyze the activity of muscle enzymes accurately, the fish were first washed with a 75% ethanol solution to remove the scales and dissected with a sharp sterile scalpel; then, collected the dorsal muscle was finally collected. The activities of the total antioxidant capacity (T-AOC), superoxide dismutase (SOD), glutathione peroxidase (GSH-Px), and catalase (CAT) and the content of malondialdehyde (MDA) and protein carbonyl (PC) in the muscle were determined according to Li [23].

T-AOC was determined using the ABTS method [23]. The absorbance was read at 405 nm using a microplate reader. T-AOC activity was calculated using the curve formula.

The xanthine and xanthine oxidase reaction system generates superoxide anions (O_2_^•−^). O_2_^•−^ can react with (water-soluble tetrazolium salt-1) WST-1 to produce a water-soluble yellow formazan, which has absorbance at 450 nm. SOD can eliminate O_2_^•−^, thereby inhibiting the formation of formazan [24].

Glutathione peroxidase (GSH-PX) facilitates the reaction between hydrogen peroxide (H_2_O_2_) and reduced glutathione (GSH) to produce water (H_2_O) and oxidized glutathione (GSSG). The activity of GSH-PX can be expressed as the rate of the reaction catalyzing GSH. In this process, GSH reacts with 5,5′-dithiobis (2-nitrobenzoic acid) to form a stable yellow anion of 5-thio-2-nitrobenzoic acid, and the absorbance at 412 nm can be measured to calculate the amount of GSH [17].

The reaction of catalase breaking down H_2_O_2_ can be rapidly stopped by adding ammonium molybdate. The remaining H_2_O_2_ reacts with ammonium molybdate to produce a light-yellow complex. The change in absorbance at 405 nm can be measured to calculate the activity of CAT [25].

Malondialdehyde (MDA), a degradation product of lipid peroxides, can condense with thiobarbituric acid (TBA) to form a red product, which has a maximum absorbance peak at 532 nm [26].

The sample was treated with 2,4-dinitrophenylhydrazine (DNPH) and dissolved in HCl. After a complete reaction, trichloroacetic acid was added and left on ice for 15 min, followed by centrifugation to obtain the protein precipitate. The protein precipitate was subsequently washed 3 times with ethanol–ethyl acetate (1:1, *v*:*v*) to remove unreacted DNPH. The final protein pellet was dissolved in guanidine hydrochloride and incubated at 37 °C for 10 min. The absorbance at 367 nm was corrected for the absorbance in the HCl blank, and the nanomolar concentration of carbonyl derivative per milligram protein was calculated using the extinction coefficient of 22,000 M^−1^·cm^−1^ [8].

### 2.6. Muscle Histological Analysis

To understand the effects of oxidized soybean meal and oxidized soybean oil on the muscle histology of experimental fish, muscle tissues from *Megalobrama amblycephala* were examined using H&E, PAS, and Masson staining. The staining was performed following the method described by Cai [27]. Four fish were selected from each group to prepare slices. We acquired muscle images from muscle sections using a panoramic scanner (Aperio VERSA 8, Leica, Weitzlar, Germany). The muscle fiber diameter was calculated by measuring 100 white muscle fibers from each sample per group using the smallest diameter method [28]. Muscle fiber density (N/mm^2^) was defined as the proportion of the total number of fibers in the entire cross-sectional area. The area of collagen and glycogen staining was quantified by ImageJ 2 pro launcher software (National Institutes of Health, Bethesda, MD, USA).

### 2.7. Determination of Flesh Quality Parameters

#### 2.7.1. Texture Assay

According to the description provided by Hixson [29], muscle texture properties were determined by a texture analyzer (TA.XT Plus, Stable Micro Systems, Surrey, U.K.) and a 50 mm diameter aluminum plate. The assay was set to a pre-test speed of 5 mm/s, a test speed of 1 mm/s, and a post-test speed of 5 mm/s, with a deformity variable of 50% of the muscle thickness and 2 presses of 60 s each for each sample.

#### 2.7.2. Myoglobin Content Assay

Myoglobin was determined by referring to the method described by Wu [30] with minor modifications. First, 1 g of chopped muscle and 5 mL of pre-cooled phosphate buffer (pH 6.8, 40 mmol/L, 4 °C) were mixed thoroughly and homogenized for 15 s. Then, samples were centrifuged at 4 °C, 8000 rpm, for half an hour. The supernatant obtained by centrifugation was filtered through 3 μm filter paper. The absorbance of the filtrate at 525, 545, 565, and 572 nm was determined, setting the phosphate buffer as a blank. The relative concentration of Mb derivatives was calculated using the following equations:

DeoMb = (0.369R1 + 1.140R2 − 0.941R3 + 0.015) × 100

OxyMb = (0.882R1 − 1.267R2 + 0.809R3 − 0.361) × 100

MetMb = (−2.514R1 + 0.777R2 + 0.800R3 + 1.098) × 100

where R1 = A572/A525, R2 = A565/A525, and R3 = A545/A525.

#### 2.7.3. Cooking Loss

Cooking loss was assessed as described by Wang [31]. A fresh muscle sample, with its weight noted as W0, was taken and placed in a water bath at 85 °C until the center temperature reached 75 °C. The water on the surface of the sample was drained using filter paper, and the weight was noted as W1. The formula for cooking loss was as follows:

Cooking loss (%) = (W0 − W1)/W0 × 100

#### 2.7.4. Myofibril Fragmentation Index (MFI)

MFI was determined by referencing the method described by Lv [32] with minor modifications. Muscle samples (2 g) were placed in 3 mL of a mixture of cold sucrose (0.24 M) and potassium chloride (0.02 M) and homogenized for 2 min. The homogenate was then filtered through a 250 µm sieve. The residue and sieve were blotted with absorbent paper while it was being weighed. The myofibril fragmentation index was calculated as follows: Myofibril fragmentation index (%) = 100 × (the weight of residue and sifter − the weight of sifter)/initial sample weight.

### 2.8. Muscle Amino Acid Analysis

Muscle amino acid profiles were determined according to the method described by López [33]. Muscle samples were degreased and then hydrolyzed in vacuum-sealed tubes with 6 N HCl at 110 °C for 24 h. The HCL was then removed with nitrogen. The solids were dissolved by ultrasonication with 0.1 N HCL and filtered through 0.22 μm polyethersulfone ultrafiltration membranes. Finally, the amino acid content was determined using an amino acid automatic analyzer (Hitachi L-8900, Tokyo, Japan). DAAs: delicious amino acids [34]; EAAs: essential amino acids; NEAAs: nonessential amino acids; TAAs: sum of total amino acids. The classification and calculation of amino acids were performed using the following formula:

DAA = Glu + Ala + Asp + Gly + Arg

EAA = Lys + Met + Thr + Val + Leu + Ile + Phe + Try + His + Arg

NEAA = Glu + Asp + Ala + Ser + Tyr + Pro + Gly + Cys

TAA = EAA + NEAA

### 2.9. Muscle Fatty Acid Analysis

Muscle fatty acid profiles were determined according to the method described by Wang [35]. The muscle samples were placed in a chloroform–methanol solution. Then, the extracted lipids were placed in 0.5 M sodium hydroxide methanol solution and heated in a 60 °C water bath to dissolve the oil droplets completely. After cooling, the extracted lipids were placed in a 25% BF_3_ methanol solution for methyl esterification. Fatty acid profiles were determined by gas chromatography. SFAs: saturated fatty acids; MUFAs: monounsaturated fatty acids; PUFAs: polyunsaturated fatty acids; ω-6/ω-3: the ratio of ω-6 PUFAs to ω-3 PUFAs.

### 2.10. Statistical Analysis

The significant difference between each parameter and two given groups was tested using the independent-samples *t*-test. All data were analyzed using the Statistical Package for the Social Sciences (SPSS) 23.0 software (IBM Crop., Armonk, NY, USA). Notes: # or ##, *p* < 0.05 or *p* < 0.01 between CON within OSM; * or **, *p* < 0.05 or *p* < 0.01 between OSM and OSO; $ or $$, *p* < 0.05 or *p* < 0.01 between CON and OSO. The data are presented as mean ± SEM (n = 4).

## 3. Results

### 3.1. Influences of OSM and OSO on Growth Performance and the Survival Rate

**Figure 1:**
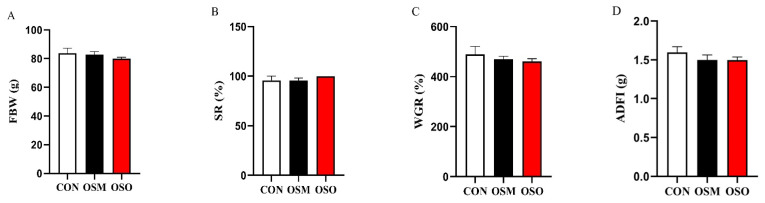
Influences of OSM and OSO on the growth performance and survival rate of blunt snout bream. (**A**) Final body weight (FBW, g). (**B**) Survival rate (SR, %). (**C**) Weight gain rate (WGR, %). (**D**) Average daily feed intake (ADFI, g).

The effects of OSM and OSO on the growth performance and survival rate of fish are shown in Figure 1. During the 8-week feeding trial period, OSM and OSO did not affect the growth performance and survival rate compared to the CON group (*p* > 0.05).

### 3.2. Influences of OSM and OSO on the Muscle Chemical Composition

**Figure 2:**
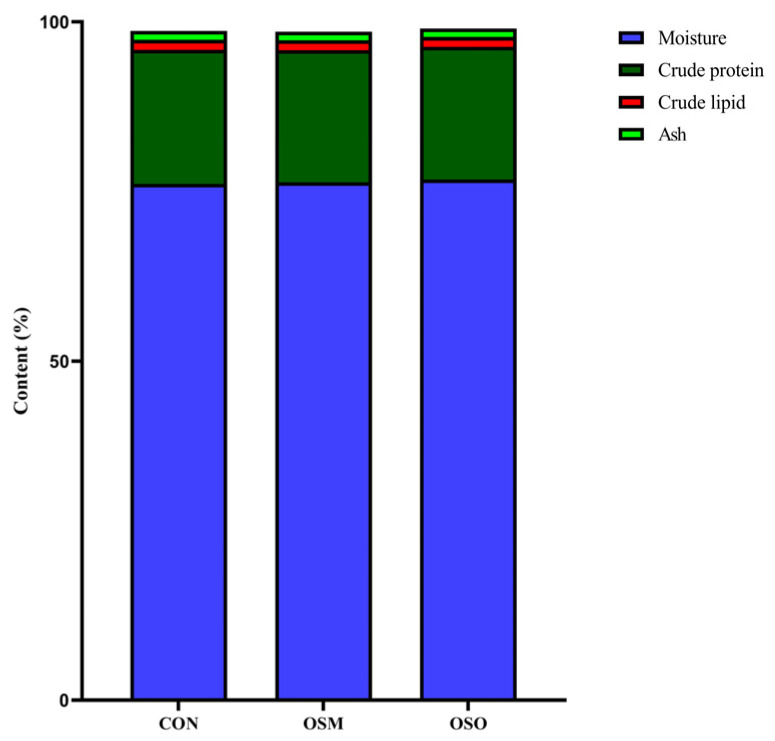
Influences of OSM and OSO on the muscle chemical composition of blunt snout bream.

The effects of OSM and OSO on muscle chemical composition are shown in Figure 2. Compared to the CON group, OSM and OSO did not affect muscle moisture, crude protein, crude fat, or ash (*p* > 0.05).

### 3.3. Influences of OSM and OSO on Muscle Oxidative Stability

**Figure 3:**
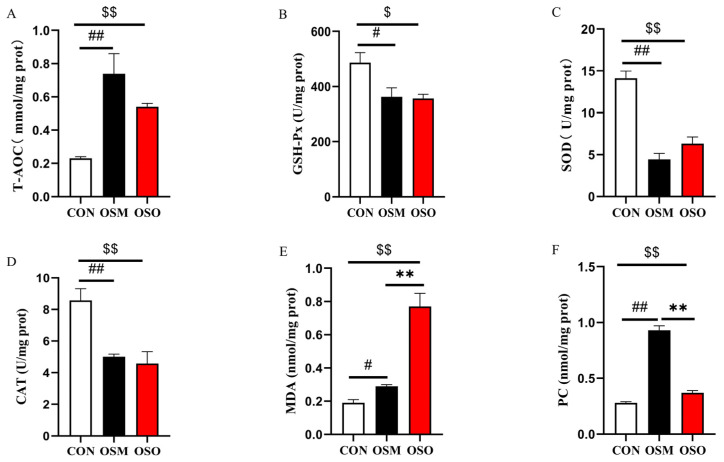
Influences of OSM and OSO on the muscle antioxidant activities of blunt snout bream. (**A**) Total antioxidant capacity (T-AOC, mmol/mg prot). (**B**) Glutathione peroxidase (GSH-Px, U/mg prot). (**C**) Superoxide dismutase (SOD, U/mg prot). (**D**) Catalase (CAT, U/mg prot). (**E**) Malondialdehyde (MDA, nmol/mg prot). (**F**) Protein carbonyl (PC, nmol/mg prot). Notes: # or ##, *p* < 0.05 or *p* < 0.01 between CON within OSM; **, *p* < 0.01 between OSM and OSO; $ or $$, *p* < 0.05 or *p* < 0.01 between CON and OSO. The data are presented as mean ± SEM (n = 4).

The effects of OSM and OSO on muscle antioxidant parameters and oxidative status are presented in Figure 3. OSM and OSO significantly increased the T-AOC capacity and the content of MDA and PC in muscle (*p* < 0.05). But they reduced the activities of GSH-Px, SOD, and CAT compared with the CON group (*p* < 0.05).

### 3.4. Influences of OSM and OSO on the Muscle Histological Structure

**Figure 4:**
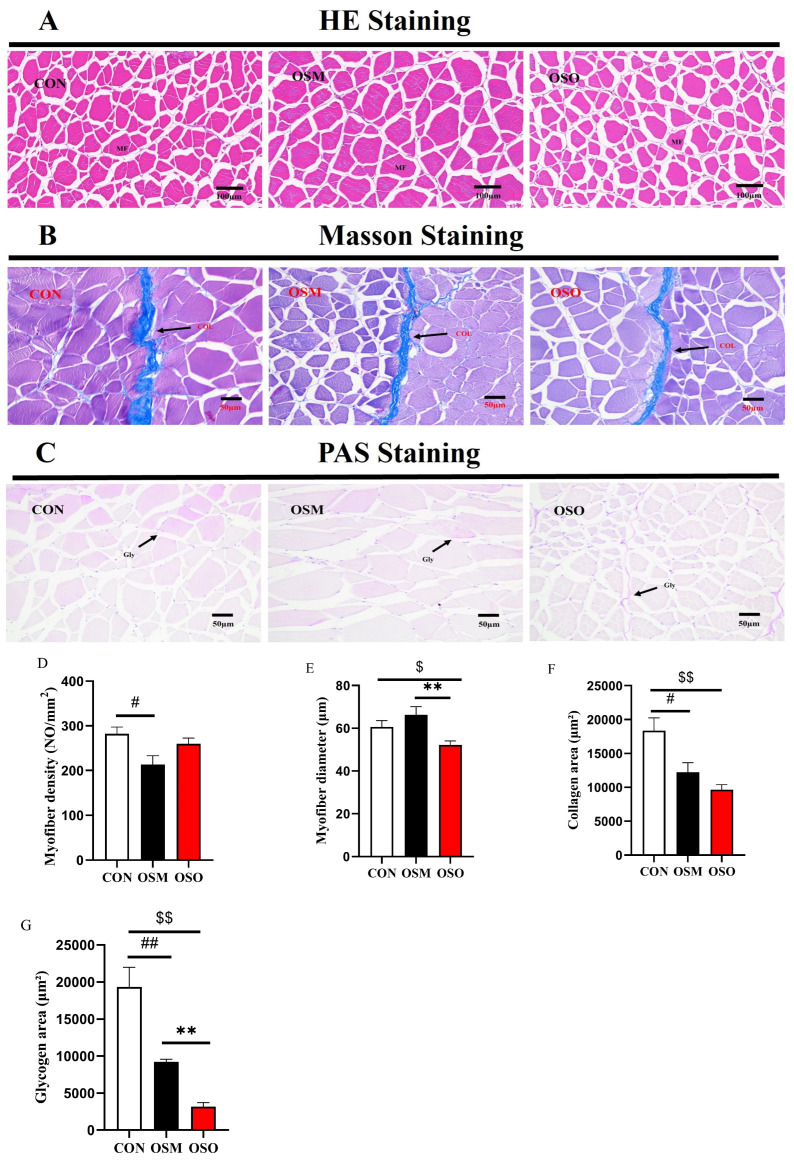
Influences of OSM and OSO on the histological analysis of blunt snout bream. (**A**) Histological observations of the dorsal muscle stained with H&E (magnification 200×; scale bars, 100 μm). (**B**) Histological observations of a dorsal muscle stained with Masson’s trichrome (magnification 200×; scale bars, 50 μm). (**C**) Histological observations of a dorsal muscle stained with PAS (magnification 200×; scale bars, 50 μm). (**D**,**E**) Quantitative graph of H&E staining. (**F**) Quantitative graph of Masson trichrome staining. (**G**) Quantitative graph of PAS staining. MF: myofiber; COL: collagen; Gly: glycogen. Notes: # or ##, *p* < 0.05 or *p* < 0.01 between CON within OSM; **, *p* < 0.01 between OSM and OSO; $ or $$, *p* < 0.05 or *p* < 0.01 between CON and OSO. The data are presented as mean ± SEM (n = 4).

The results for the histologically stained sections of muscles are presented in Figure 4. The HE staining of a muscle is shown in Figure 4A. By performing a semiquantitative analysis of the myofiber density and myofiber diameter in all groups of sections, OSM was observed to significantly decrease the myofiber density (Figure 4D) (*p* < 0.05). It is notable that OSO significantly reduced the myofiber diameter compared with the other groups (Figure 4E) (*p* < 0.05). The Masson staining of a muscle is shown in Figure 4B. Compared with the CON group, OSM and OSO significantly reduced the collagen area (Figure 4F) (*p* < 0.05). The PAS staining of a muscle is shown in Figure 4C. Similar to the Masson staining results, OSM and OSO significantly reduced the glycogen area (Figure 4G) (*p* < 0.05).

### 3.5. Influences of OSM and OSO on Muscle Quality Parameters

**Figure 5:**
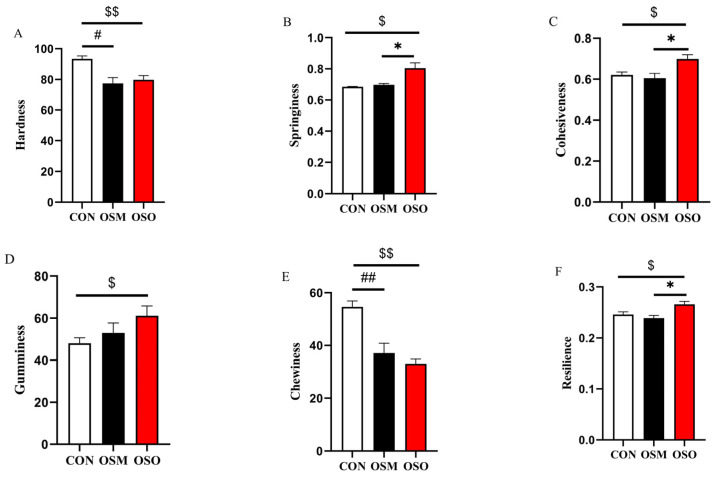

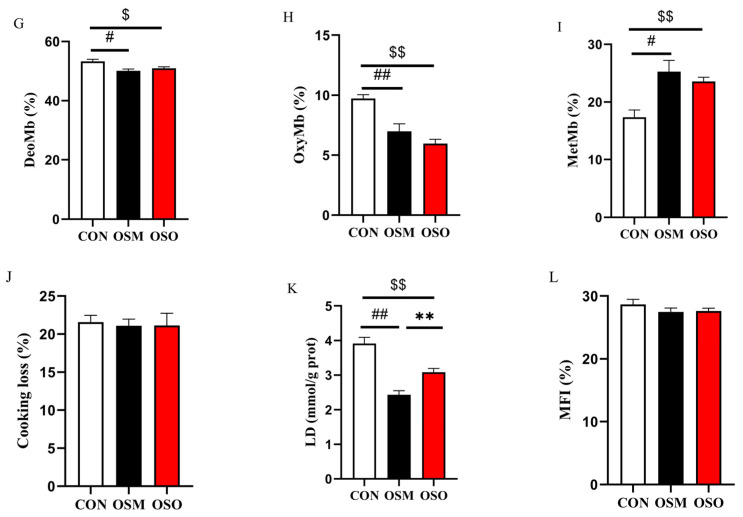
Influences of OSM and OSO on the muscle textural properties, myoglobin, cooking loss, and MFI of blunt snout bream. (**A**) Hardness. (**B**) Springiness. (**C**) Cohesiveness. (**D**) Gumminess. (**E**) Chewiness. (**F**) Resilience. (**G**) Deoxymyoglobin (DeoMb, %). (**H**) Oxymyoglobin (OxyMb, %). (**I**) Metmyoglobin (MetMb, %). (**J**) Cooking loss (%). (**K**) Lactic acid (LD, mmol/g prot). (**L**) Muscle fragmentation index (MFI, %). Notes: # or ##, *p* < 0.05 or *p* < 0.01 between CON within OSM; * or **, *p* < 0.05 or *p* < 0.01 between OSM and OSO; $ or $$, *p* < 0.05 or *p* < 0.01 between CON and OSO. The data are presented as mean ± SEM (n = 4).

The muscle texture parameters are shown in Figure 5A–F. OSM and OSO significantly decreased the hardness and chewiness (*p* < 0.05). Compared with the CON and OSM, OSO was observed to significantly increase the springiness, cohesiveness, and resilience (*p* < 0.05). The relative concentrations of Mb derivatives are presented in Figure 5G–I. OSM and OSO significantly decreased the relative concentration of DeoMb and OxyMb, while it elevated MetMb (*p* < 0.05). Compared with the CON group, OSM and OSO significantly reduced the content of lactic acid in the muscle (Figure 5K) (*p* < 0.05). As can be seen in Figure 5J,L, OSM and OSO did not have a significant effect on cooking loss or MFI (*p* > 0.05).

### 3.6. Influences of OSM and OSO on the Muscle Amino Acid Profile

Table 4 shows the muscle amino acid profile in blunt snout bream. Compared with CON and OSO, OSM significantly reduced the content of DAAs, EAAs, NEAAs, and TAAs (*p* < 0.05).

### 3.7. Influences of OSM and OSO on the Muscle Fatty Acid Profile

The fatty acid profile is presented in Table 5. Compared with CON, OSM significantly reduced the content of pentadecanoic acid (C15:0) and elaidic acid (C18:1n9t) (*p* < 0.05). Compared with CON and OSM, OSO significantly reduced the content of elaidic acid (C18:1n9t), linoelaidic acid (C18:2n6c), ω-6 PUFAs, PUFAs, and the ratio of ω-6/ω-3 (*p* < 0.05), while stearic acid (C18:0), γ-linolenic acid (C18:3n6), and SFAs were significantly elevated (*p* < 0.05).

## 4. Discussion

### 4.1. Antioxidant Capacity

In this study, we found that OSM and OSO increased muscle T-AOC activity, MDA, and PC content and decreased the activity of SOD, CAT, and GSH-Px. MDA is a marker of lipid peroxidation and one of the end products of PUFA peroxidation. PC is one of the products of protein oxidation [36]. These results suggest that the antioxidant system of the fish is disturbed, and the organism depletes its own antioxidant enzymes to eliminate the damage caused by oxidative stress. T-AOC is the total antioxidant capacity of a system that contains antioxidant enzymes and other antioxidants. Although the fish body’s antioxidant enzyme activity was reduced, the body increased its own total antioxidant capacity in response to the imbalance in the redox state brought about by oxidized soybean meal or oxidized soybean oil. It has been demonstrated that oxidized protein and oxidized fat trigger oxidative stress in many animals, such as broiler chickens [8] and rats [37]. Zhou [11] also demonstrated that oxidized proteins or oxidized fats significantly alter the antioxidant defense system of the body. These results are consistent with the current findings. Oxidative stress is also one of the main reasons for the decline in flesh quality, as fish muscle is rich in PUFAs. The decrease in antioxidant capacity may result in the oxidation of membrane phospholipids, which in turn makes biofilms less fluid, disrupts normal membrane structure and function, and impairs the structural integrity of muscles [38]. All of these can adversely affect flesh quality. Therefore, the next step in the process was to continue the exploration related to flesh quality.

### 4.2. Flesh Quality

Color, tenderness, pH, and muscle texture are commonly used to assess flesh quality [39]. Muscle color is an important indicator for assessing sensory quality, which determines the first impression of a muscle and is an important factor influencing consumers’ purchasing decisions [40]. In this study, OSM and OSO decreased the content of DeoMb and OxyMb and increased the content of MetMb. Myoglobin is a metalloprotein that mainly exists in three states, i.e., DeoMb, OxyMb, and MetMb. The content and state of myoglobin are closely related to muscle color [41]. When myoglobin is oxidized to OxyMb, the muscle takes on a bright red color. However, when improperly stored for a long time, OxyMb is oxidized to DeoMb, at which point the meat’s brightness value decreases and takes on a brown color [42]. Protein and lipid oxidation contributes to accelerated ferric myoglobin oxidation [43]. In this study, we found that increased MDA and PC contents in the muscle were accompanied by increased MetMb content. A study on channel catfish *Ictalurus punctatus* found that oxidized soybean oil also significantly increased the yellowness (b*) value of the skin and muscle [3]. It has been shown that adding antioxidants in feed improves muscle color [44].

pH is a central indicator for evaluating flesh quality. The rate and extent of muscle pH decline after animal slaughter directly affects water holding capacity (WHC) and tenderness [39]. Changes in pH are mainly related to the content of lactic acid; therefore, the content of lactic acid is commonly used to assess the pH of muscle [45]. Anaerobic glycolysis is the main metabolic pathway in animals after slaughter, which leads to the accumulation of lactic acid in muscles, thus decreasing the pH value [46]. In the present study, we found that OSM and OSO reduced the lactic acid content in the muscle. We speculate that the decrease in glycogen content may have reduced the substrate for the reaction of anaerobic glycolysis, thus reducing lactic acid production [47]. This helps improve the muscle’s WHC, such as cooking loss [48]. However, an imbalance in redox reactions in the muscle can lead to cell membrane rupture, reducing the muscle’s WHC [49]. This may also be the reason why cooking loss did not change in this study.

Tenderness is one of the most important indicators for evaluating flesh quality. MFI and meat tenderness are positively correlated [50]. Lower pH is detrimental to flesh tenderization, as it leads to a decrease in calpain activity [51]. However, the present study found that OSM and OSO reduced the muscle lactic acid content but did not affect MFI. Tenderness is related to collagen, sarcomere length, and pH [52,53]. Qi [54] found that altering the number of fibers and the strength of connective tissue in muscles can affect tenderness. The enzymatic degradation of cytoskeletal proteins is considered a key factor in the changes in tenderness during muscle maturation [55]. In this study, OSM and OSO reduced the collagen content and total amino acid content in the muscle. Additionally, the occurrence of oxidative stress in a muscle can impact the activity of calpain and the structure of substrate proteins. In summary, MFI is influenced by various factors, and the effects of OSM and OSO on meat quality require further investigation.

The texture of muscles also has a direct impact on the consumer’s taste, with muscles that have greater firmness and chewiness generally being more popular among consumers [56]. Better texture also facilitates the processing of fish into high-quality aquatic products. Postembryonic muscle growth in most vertebrate fish differs from that of terrestrial animals, showing great plasticity to the environment. While terrestrial animals have only hypertrophy of muscle fibers after birth, hyperplasia and hypertrophy of muscle fibers exist in most of the life cycle of fish species [57,58]. This also gives aquaculture a greater advantage in the production of meat foods compared with the livestock industry. Textural characterization is a reliable and commonly used method to evaluate the sensory of muscles by simulating human chewing [59]. OSM and OSO reduced the hardness and chewiness of the muscle. This result suggests that OSM and OSO adversely affected the muscle texture. Myofibers, as the basic unit of muscles, are one of the most important factors affecting the texture of fish muscle. Previous studies have found that muscles with greater myofiber density have greater textural properties such as hardness and chewiness [60]. In the present study, we observed that OSM and OSO significantly reduced the density of muscle fibers. This may be one of the reasons for the decrease in muscle hardness and chewiness. In addition, a decrease in collagen content also affects muscle hardness [61]. Nutritional content is an important part of assessing the value of fish as a high-value food. For this reason, next, we continue to discuss the effects of OSM and OSO on muscle nutrient composition.

### 4.3. Muscle Chemical Composition

The chemical composition of fish muscle, which is mainly moisture, proteins, lipids, minerals, and glycogen, reflects the nutritional value and physiological status of fish [9]. Detailed information on these components is required by fish nutritionists, feed producers, aquaculture practitioners, food manufacturers, human health nutritionists, and consumers. In this study, the results showed that both OSM and OSO reduced the glycogen and collagen content of the muscle but had no effect on the crude protein, crude fat, moisture, or ash. Fish are preferred for their “high protein, low fat” characteristics, and the texture and flavor of their muscles directly affect whether consumers will make repeat purchases. Consumers generally prefer a firmer, more flavorful fish muscle. Many of the aroma compounds in muscle are produced by the breakdown of glycogen, and an increase in glycogen content contributes to the flavor of muscle [62]. The collagen content is closely related to muscle texture, and it facilitates muscle firmness and improves muscle hardness [63]. OSM and OSO not only directly reduce the nutritional value of muscles but also cause damage to the texture and flavor of muscles. Chi [15] found that oxidized soybean oil significantly increased the hypoxanthine (Hx) and hypoxanthin riboside (HxR) content in the muscle of the blunt snout bream. A study found that the addition of oxidized soybean oil at a ratio of 60 g/kg to the diet reduced the crude fat content of channel catfish *Ictalurus punctatus* muscle, which is not consistent with the present study [3]. We speculate that the possible reason for this is the difference in the percentage of addition, which was 4.19% of oxidized soybean oil in the present study. In addition, the present study found for the first time that oxidized soybean meal adversely effects the nutritional value and flavor of muscles.

### 4.4. Muscle Amino Acid Profile

Amino acids are important indicators in assessing the nutritional value of meat foods [64]. For this reason, we further investigated the effects of OSM and OSO on the amino acid content in the muscle. The results showed that OSM reduced the contents of EAAs, NEAAs, and TAAs in the muscle. Previous study results showed that OSM did not affect CP content in muscles, and the reason for this difference may be that the CP content was calculated using the nitrogen content (CP = nitrogen content/16%). However, other nitrogen-containing substances, such as phospholipids, exist in muscles in addition to proteins, and CP is not exactly equivalent to TAAs. Essential amino acids must be obtained from the diet to meet the body’s needs. Therefore, when any EAA is in excess or deficient, it will affect the availability of protein and the normal growth and development of the body. It is clear that OSM reduces the nutritional value and potential consumption value of fish muscle. In addition to their nutritional value, some amino acids are capable of directly imparting flavor, such as the following sweet amino acids: Gly, Ala, Pro, Ser, and Thr, and the following flavor amino acids: Asp, Glu, Gly, Ala, and Arg, or reacting with other substances to produce flavor substances in muscles. Methionine can react with xylose to produce 3-(methylthio) propionaldehyde via a Maillard reaction and has potato, onion, and meat flavors. The reaction of leucine produces 3-(methyl) butyraldehyde, which has a peachy and fatty aroma [65]. In this study, OSM significantly reduced the amount of these flavor-presenting amino acids in the muscle. This can adversely affect the flavor and overall level of the muscle. It has been shown that the amino acid composition of muscle varies with diet composition [66]. For this reason, we hypothesized that after the oxidation of soybean meal, it reduces the amino acid content, which in turn leads to an imbalance in amino acids in the diet. In addition, exogenous stimuli also lead to the accumulation of ROS in muscles, and free radicals are more likely to react with residues of amino acids, leading to their depletion and reducing the nutritional value of muscle foods.

At the same time, the levels of certain amino acids are closely related to normal metabolism in the body. For instance, arginine (Arg) can promote the immune system’s secretion of endogenous substances like leukocytes and phagocytes, enhancing immunity [67]. Leucine can induce an increase in antioxidant enzyme activity, boosting the body’s antioxidant capacity [68]. Serine is a metabolic intermediate involved in the synthesis of methionine, glycine (Gly), and cysteine (Cys), playing a key role in lipid metabolism, the immune system, and the central nervous system [67]. As a result, the decrease in EAA, NEAA, and TAA levels in muscles not only reduces the nutritional value of fish muscles but also negatively impacts the health of the fish body. Fish have a higher protein content than most terrestrial meats and exhibit high digestibility, making them one of the most nutritious components of the human diet [64]. Research on the relationship between fish protein and various human diseases, such as inflammation, metabolic syndrome, and insulin resistance, has received increasing attention [69]. Madani [70] suggested that dietary sardine protein may be a means of preventing insulin resistance. Dort [71] found that cod protein promotes post-injury skeletal muscle growth and regeneration better than peanut protein and casein. These results indicate that consuming fish has positive effects on human health. Therefore, the reduction in amino acid content in fish muscle due to OSM not only adversely affects the health of the fish itself but also diminishes the nutritional value for consumers and negatively impacts the prevention of potentially related diseases.

### 4.5. Muscle Fatty Acid Profile

Fatty acids in fish not only reflect their physiological condition but also their nutritional and potential health value. The results of the present study showed that OSM did not affect the fatty acid composition of the muscle compared with CON; however, OSO increased the percentage of SFAs and decreased the percentage of PUFAs and the ratio of ω-6/ω-3 in the muscle. ω-3 PUFAs, the main constituents of which are DHA and EPA, have various physiological functions in fish, such as maintaining cell membrane integrity, inhibiting inflammatory responses, regulating glucose–lipid metabolism, etc. [72]. It has been found that inflammatory cells usually contain high levels of ω-6 PUFAs, so the intake of large amounts of ω-6 PUFAs, especially arachidonic acid, may promote the inflammatory process [73]. It is more intuitive to evaluate the nutritional value of fish using the ratio of ω-6/ω-3; the higher the ratio, the lower the nutritional value [74]. Diets containing excessive amounts of SFAs have been strongly associated with the development of cardiovascular disease [75]. Thus, the results of the current study suggest that OSO reduced the nutritional value of fatty acids in the muscle, which in turn may also pose a health hazard to potential consumers. Studies on fish have shown that the fatty acid composition of muscles varies the fatty acid composition of the diet [76]. We speculate that the reason for this may be that as the oxidation of soybean oil reduces the content of PUFAs, they become peroxides and, eventually, suffer fragmentation into secondary oxidation compounds, which in turn disrupts the balance of polyunsaturated fatty acids in the diet. In addition, PUFAs are the main substrates for oxidative reactions, and an increase in SFAs may be due to the decrease in PUFA as a result of oxidative reactions. Muscles have shown higher levels of DHA and less EPA compared to the liver [77]. However, the deposition of individual fatty acids in muscle tissue may be regulated by different mechanisms, such as preferential binding, β-oxidation, and fatty acid desaturation processes [78]. Therefore, further studies are needed to explain these results. However, current studies on the effect of oxidized oils on muscle fatty acid composition vary widely. This may be related to factors such as animal species, animal size, degree of lipid oxidation, and differences in test formulations and feeding management.

Oxidized soybean meal and oxidized soybean oil have obvious negative effects on fish. Therefore, preventing or reducing oxidation is beneficial for the sustainable development of the aquaculture industry. Soybean meal contains a high level of crude protein, which is prone to oxidation when exposed to oxygen, light, high temperature, high humidity, and other oxidative conditions. The oxidation of proteins in soybean meal can lead to structural changes, thus reducing its nutritional value and digestibility [79]. Additionally, soybean meal has a strong ability to absorb moisture [80]. Soybean oil, rich in polyunsaturated fatty acids (PUFAs), also undergoes oxidation under exposure to oxidizing agents or oxidative conditions, which not only diminishes its nutritional value but also generates a series of harmful substances, resulting in color changes and off-flavors [81]. As one of the most traded feed ingredients, the shipping, land transportation, and storage processes undoubtedly extend the time from production to use [80,82]. These factors facilitate the oxidation of both soybean meal and soybean oil. To minimize unnecessary losses, it is crucial to reduce the occurrence of oxidation in soybean meal and soybean oil. First, it is necessary to improve the production methods of soybean meal by reducing the production of high-temperature pressed soybean meal. Second, during transportation, it is important to adopt airtight packaging for soybean meal and soybean oil to minimize exposure to light and oxygen. Finally, improving storage conditions to maintain low temperatures and dryness is vital.

## 5. Conclusions

In the current study, neither oxidized soybean meal (OSM) nor oxidized soybean oil (OSO) affected the growth performance of blunt snout bream. However, both OSM and OSO significantly reduced the muscle antioxidant capacity and negatively impacted the flesh color and muscle texture of blunt snout bream. In addition, OSM reduced the content of essential amino acids (EAAs), non-essential amino acids (NEAAs), delicious amino acids (DAAs), and total amino acids (TAAs) in the muscle, while OSO decreased the content of polyunsaturated fatty acids (PUFAs) and increased the content of saturated fatty acids (SFAs) in the muscle.

## Figures and Tables

**Table 1:** Characteristics of the experimental soybean meal.

	Fresh Soybean Meal	Oxidized Soybean Meal
Protein carbonyl (nmol/mg of protein)	3.77	11.81
Free sulfhydryl (nmol/mg of protein)	10.01	3.71

**Table 2:** Characteristics of the experimental soybean oil.

	Fresh Soybean Oil	Oxidized Soybean Oil
Acid value (g KOH/kg)	0.34	3.77
Peroxides (meq/kg)	2.27	463.75

**Table 3:** Composition of experimental basal diets.

Ingredients (%)	CON	OSM	OSO
Fish meal	5.00	5.00	5.00
Fresh soybean meal	30.00	0.00	30.00
Oxidized soybean meal	0.00	30.00	0.00
Cottonseed meal	14.39	14.39	14.39
Rapeseed meal	5.00	5.00	5.00
Wheat middling	25.00	25.00	25.00
Wheat bran	12.62	12.62	12.62
Fresh soybean oil	4.19	4.19	0.00
Oxidized soybean oil	0.00	0.00	4.19
Premix *	1.00	1.00	1.00
Calcium biphosphate	1.80	1.80	1.80
Sodium chloride	0.50	0.50	0.50
Ethoxyquin	0.50	0.50	0.50
Total	100.00	100.00	100.00
Nutrient content (% dry matter)			
Crude protein	31.00	31.00	31.00
Crude fat	6.00	6.00	6.00

* Premix supplied the following minerals (g/kg of diet) and vitamins (IU or mg/kg of diet): CuSO_4_⋅5H_2_O, 2.0 g; FeSO_4_⋅7H_2_O, 25 g; ZnSO_4_⋅7H_2_O, 22 g; MnSO_4_⋅4H_2_O, 7 g; Na_2_SeO_3_, 0.04 g; KI, 0.026 g; CoCl_2_⋅6H_2_O, 0.1 g; vitamin A, 900,000 IU; vitamin D, 200,000 IU; vitamin E, 4500 mg; vitamin K_3_, 220 mg; vitamin B_1_, 320 mg; vitamin B_2_, 1090 mg; vitamin B_5_, 2000 mg; vitamin B_6_, 500 mg; vitamin B_12_, 1.6 mg; vitamin C, 5000 mg; pantothenate, 1000 mg; folic acid, 165 mg; and choline, 60,000 mg.

**Table 4:** Muscle amino acid profile of blunt snout bream.

	CON	OSM	OSO
Asp	2.06 ± 0.02 ^##^	1.98 ± 0.01 **	2.09 ± 0.01
Thr	0.89 ± 0.01 ^#^	0.86 ± 0.001 **	0.9 ± 0.01
Ser	0.82 ± 0.01 ^##^	0.79 ± 0.01 **	0.83 ± 0.01
Glu	3.13 ± 0.03 ^##^	3 ± 0.01 **	3.15 ± 0.02
Gly	0.92 ± 0.01 ^#^	0.87 ± 0.01 **	0.94 ± 0.01
Ala	1.16 ± 0.01 ^##^	1.11 ± 0.01 **	1.19 ± 0.01
Cys	0.16 ± 0.01 ^#^	0.19 ± 0.01 *	0.21 ± 0.001 ^$$^
Val	0.95 ± 0.01 ^##^	0.92 ± 0.001 **	0.96 ± 0.01
Met	0.65 ± 0.001 ^##^	0.62 ± 0.001 **	0.66 ± 0.001
Ile	0.92 ± 0.01 ^##^	0.87 ± 0.001 **	0.93 ± 0.01
Leu	1.64 ± 0.01 ^##^	1.57 ± 0.01 **	1.66 ± 0.01
Tyr	0.69 ± 0.01 ^##^	0.66 ± 0.001 **	0.7 ± 0.01
Phe	0.85 ± 0.01 ^#^	0.81 ± 0.001 **	0.87 ± 0.01
Lys	1.92 ± 0.02 ^##^	1.84 ± 0.01 **	1.94 ± 0.01
His	0.61 ± 0.01	0.59 ± 0.01 *	0.62 ± 0.01
Arg	1.17 ± 0.01 ^##^	1.13 ± 0.01 **	1.18 ± 0.01
Pro	0.58 ± 0.01 ^#^	0.56 ± 0.01 **	0.59 ± 0.001
EAAs	9.61 ± 0.09 ^##^	9.21 ± 0.03 **	9.73 ± 0.06
NEAAs	9.54 ± 0.08 ^##^	9.16 ± 0.05 **	9.69 ± 0.07
DAAs	8.1 ± 0.08 ^##^	7.75 ± 0.04 **	8.2 ± 0.05
TAAs	19.15 ± 0.17 ^##^	18.37 ± 0.08 **	19.42 ± 0.13

Notes: # or ##, *p* < 0.05 or *p* < 0.01 between CON within OSM; * or **, *p* < 0.05 or *p* < 0.01 between OSM and OSO; $$, *p* < 0.01 between CON and OSO. The data are presented as mean ± SEM (n = 4).

**Table 5:** Muscle fatty acid profile of blunt snout bream (%).

	CON	OSM	OSO
C14:0	1.14 ± 0.04	1.06 ± 0.04	1.13 ± 0.09
C15:0	0.39 ± 0.02 ^##^	0.24 ± 0.01 **	0.25 ± 0.02
C16:0	17.33 ± 2.75	16.31 ± 0.62	24.26 ± 1.37 ^$$^
C16:1	1.15 ± 0.18	1.25 ± 0.11	1.09 ± 0.09
C17:0	0.46 ± 0.03	0.52 ± 0.04	0.55 ± 0.04
C18:0	14.98 ± 0.29	16.25 ± 0.55 **	18.07 ± 0.42 ^$^
C18:1n9t	1.35 ± 0.02 ^#^	1 ± 0.13 **	0.65 ± 0.03 ^$^
C18:1n9c	16.86 ± 0.66	16.3 ± 0.27	14.76 ± 1.05
C18:2n6t	0.57 ± 0.04	0.5 ± 0.03	0.6 ± 0.08
C18:2n6c	25.2 ± 1.32	24.72 ± 0.46 **	16.04 ± 0.39 ^$$^
C20:0	0.24 ± 0.02	0.27 ± 0.02	0.29 ± 0.02
C18:3n6	0.32 ± 0.02	0.41 ± 0.05 **	0.69 ± 0.03 ^$$^
C20:1	0.4 ± 0.1	0.39 ± 0.07	0.51 ± 0.06
C18:3n3	3.56 ± 0.08	3.69 ± 0.24 *	3.26 ± 0.05
C20:2	3.21 ± 0.44	3.79 ± 0.19	3.11 ± 0.16 ^$^
C22:0	0.2 ± 0	0.21 ± 0.01	0.22 ± 0.01
C20:3n6	1.78 ± 0.2	1.91 ± 0.08	2.09 ± 0.08
C22:1n9	0.53 ± 0.09	0.65 ± 0.02	0.52 ± 0.08
C20:4n6	3.32 ± 0.57	3.09 ± 0.23	3.78 ± 0.39
C24:0	0.63 ± 0.14	0.73 ± 0.08	0.79 ± 0.03
C20:5n3	0.62 ± 0.02	0.59 ± 0.03	0.59 ± 0.04
C22:6n3	5.8 ± 0.64	6.16 ± 0.39	6.76 ± 0.43
SFAs	35.35 ± 2.74	35.58 ± 0.12 *	45.55 ± 1.05 ^$$^
MUFAs	20.28 ± 0.75	19.59 ± 0.28	17.53 ± 1.1
ω-3 PUFAs	7.98 ± 0.64	8.43 ± 0.32	8.61 ± 0.38
ω-6 PUFAs	31.18 ± 2	30.62 ± 0.35 **	23.2 ± 0.77 ^$$^
ω-6/ω-3	3.94 ± 0.23	3.65 ± 0.18 **	2.7 ± 0.07 ^$$^
PUFAs	42.37 ± 2.45	42.84 ± 0.19 *	34.92 ± 1.17 ^$$^

Notes: fatty acids were detected here. Notes: # or ##, *p* < 0.05 or *p* < 0.01 between CON within OSM; * or **, *p* < 0.05 or *p* < 0.01 between OSM and OSO; $ or $$, *p* < 0.05 or *p* < 0.01 between CON and OSO. The data are presented as mean ± SEM (n = 4).

## Data Availability

The data generated during the current study are available from the first author.
